# Predictors for success in renal denervation–a single centre retrospective analysis

**DOI:** 10.1038/s41598-018-33783-3

**Published:** 2018-10-19

**Authors:** Alexander Reshetnik, Christopher Gohlisch, Christian Scheurig-Münkler, Maximilian De Bucourt, Walter Zidek, Markus Tölle, Markus van der Giet

**Affiliations:** 1 Charité – Universitätsmedizin Berlin, a corporate member of Freie Universität Berlin, Humboldt-Universität zu Berlin, and Berlin Institute of Health, Campus Benjamin Franklin, Department of Nephrology, Hindenburgdamm 30, 12203 Berlin, Germany; 2 Charité – Universitätsmedizin Berlin, a corporate member of Freie Universität Berlin, Humboldt-Universität zu Berlin, and Berlin Institute of Health, Campus Benjamin Franklin, Department of Radiology, Hindenburgdamm 30, 12203 Berlin, Germany; 3 Department for Diagnostic and Interventional Radiology and Neuroradiology, Universitaetsklinikum Augsburg, Stenglinstr. 2, 86156 Augsburg, Germany

**Keywords:** Renovascular hypertension, Vascular diseases

## Abstract

Renal denervation (RDN) is one of the most frequently used invasive methods for the treatment of arterial hypertension. However, recent randomized sham-controlled studies raised concern about the efficacy and predictability of response. We retrospectively analyzed outcomes of patients, who underwent RDN in our hypertension center between November 2010 and April 2014 and report here outcomes twelve months after procedure based on 24-hours ambulatory blood pressure monitoring. We defined ten-mm Hg decrease in office systolic blood pressure (SBP) as a cut-off for response and looked for possible predictors of this response using binary multiple regression analysis. 42 patients were included. Their mean age was 59.6 ± 9.2 years and 24% were female. Baseline office SBP and diastolic blood pressure (DBP) were 164.1 ± 20.3 and 91.8 ± 12.4 mm Hg respectively. Mean 24 h-SBP significantly decreased from 149.8 ± 13.3 mm Hg to 141.2 ± 14.6 mm Hg. Mean 24 h-DBP significantly decreased from 83.3 ± 11.7 mm Hg to 78.8 ± 11.2 mm Hg. A higher level of mean 24 h-DBP and office DBP was shown to be predictive for response in office BP and a higher level of mean 24 h-DBP for response in 24 h-SBP and 24 h-DBP. Further properly designed randomized trials are warranted to confirm this finding as well as further investigate the role of diabetes mellitus and arterial stiffness in RDN.

## Introduction

Arterial hypertension has a major disease burden. Percutaneous renal denervation (RDN) has been shown to effectively reduce blood pressure (BP) in different previous trials^1–3^. However, reported responses vary from large falls in BP to unchanged or small increase in BP even within a given study population^4^. This suggests, that specific patient characteristics may predict the response to RDN. Establishing such predictors is very important for clinicians since significant fall in BP after RDN could cause more morbidity due to hypotension, whereas poor response would not be expected to reduce cardiovascular events, which is the primary purpose of BP reduction. The randomized and sham-controlled SIMPLICITY-HTN 3 trial could not show any benefit of RDN compared to sham-group with optimal medical treatment^5^ and raised doubt about the effectiveness of RDN, though reliable data support the “proof of concept” of RDN^6,7^. Recently published sham-controlled Spyral HTN ON-MED and OFF-MED studies demonstrated clinically relevant fall in 24-hours ambulatory systolic BP (SBP) with 7.4 mm Hg after six months in the ON-MED trial and 5.5 mm Hg after three months in the OFF-MED trial^8,9^. In the sham-controlled, prospective, double-blind Radiance-HTN solo trial, where an intravascular applied ultrasound was used for renal artery nerve ablation, 24-h-SBP reduction of 5 mm Hg was observed^10^. Both studies showed a clear tendency for the effectiveness of the RDN procedure. However, the magnitude of response was unpredictable with some over-responder and only about 70–80% presenting with any BP reduction after RDN.

In the light of current data, identification of the predictors for response to RDN seems to be crucial in order to select the patients appropriately. The response should be evaluated according to change in mean 24 h-SBP and diastolic BP (DBP) obtained with ambulatory blood pressure monitoring (ABPM), due to known shortcomings of office BP^11^. Based on ABPM readings, more data are needed from a “real-world setting” dealing with reliable predictors of the response to RDN. In this retrospective analysis, we sought to identify such baseline parameters in a cohort of patients with refractory hypertension, who underwent RDN in our centre. We considered changes in 24 h-BP measured with ABPM in order to avoid shortcomings related to office BP-based assessment of treatment effect.

## Results

In total, 81 patients underwent RDN in our nephrology and hypertensiology department at Charité University Berlin, Campus Benjamin Franklin between November 2010 and April 2014. Complete demographic, clinical and procedure-related data and follow-up data including the course of BP values assessed with ambulatory blood pressure monitoring over 24 hours (24 h-ABPM) was available for 42 subjects, who were included in our retrospective analysis. According to the chosen response definition, twenty-one patients showed BP-response after RDN. Mean patient age, BMI, kidney function, amount of albuminuria, office SBP and DBP, heart rate and the number of antihypertensive medications at baseline and after twelve months did not differ significantly in both groups. In the responder group, the mean 24 h-SBP and -DBP at baseline were significantly higher compared to the non-responder group. In the non-responder group the number of patients with diabetes was significantly higher than in the responder group. Details of demographics and clinical data of the whole collective, responder and non-responder group are presented in Table 1.

**Table 1:** Demographic and clinical characteristics of the study collective (n = 42).

	Whole collective (n = 42)	Non-responder n = 21	Responder n = 21
Age, years	59.6 ± 9.2	61.4 ± 8.3	57.7 ± 9.8
Female sex, n (%)	10 (24)	5(24)	5 (24)
BMI, kg/m^2^	30.1 ± 4.6	30.4 ± 4.1	29.8 ± 5.11
Diabetes, n (%)	21 (50)	15 (71.4)	6 (28.6)*
HbA1C, %	6.3 ± 1.0	6.0 ± 0.84	6.5 ± 1.0
eGFR, ml/min/1,73 m^2^	81.3 ± 16.0	78.6 ± 18.1	84.1 ± 13.2
albuminuria, mg/g creatinine	73 ± 338	162 ± 535	14 ± 18
Isolated systolic hypertension, n(%)		12 (57.1)	6 (28.6)
coronary artery disease		6 (29)	6 (29)
Office SBP at baseline (mm Hg)	164.1 ± 20.3	163 ± 22.7	165.2 ± 18.0
Office DBP at baseline (mm Hg)	91.8 ± 12.4	89.2 ± 13.9	97.0 ± 16.1
24-h mean SBP (mm Hg)	149.8 ± 13.3	144.5 ± 13.1	155.1 ± 11.4^*^
24-h mean DBP (mm Hg)	83.3 ± 11.7	77.0 ± 10.8	89.4 ± 9.1^#^
Heart rate at baseline (bpm)	65.5 ± 10.0	64.8 ± 11.8	66.1 ± 8.1
Antihypertensives at baseline	5.5 ± 1.4	5.9 ± 1.1	5.2 ± 1.7
ACEI/ARB, n(%)	39 (92.9)	20 (95.2)	19 (90.5)
Calcium channel blocker, n(%)	35 (83.3)	19 (90.5)	16 (76.2)
Betablocker, n(%)	34 (81.0)	17 (81.0)	17 (81.0)
Aldosterone antagonist, n(%)	5 (11.9)	4 (19.0)	1 (4.8)
Thiazide, n(%)	39 (92.9)	20 (95.2)	19 (90.5)
Nitrates, n(%)	9 (21.4)	4 (19)	5 (23.8)
Central alpha2-agonists, n(%)	22 (52.4)	13 (61.9)	9 (42.9)
Direct vasodilators, n(%)	7 (9.5)	5 (23.8)	2 (9.5)
Antihypertensives at 12 months	5.1 ± 1.2^§^	5.2 ± 1.0	4.9 ± 1.4
ACEI/ARB, n(%)	38 (90.5)	20 (95.2)	18 (85.7)
Calcium channel blocker, n(%)	29 (69.0)	15 (71.4)	14 (66.7)
Betablocker, n(%)	36 (85.7)	17 (81)	19 (90.5)
Aldosterone antagonist, n(%)	5 (11.9)	3 (14.3)	2 (9.5)
Thiazide, n(%)	41 (97.6)	21 (100.0)	20 (95.2)
Nitrates, n(%)	5 (11.9)	4 (19)	1 (4.8)
Central alpha2-agonists, n(%)	18 (42.9)	9 (42.9)	9 (42.9)
Direct vasodilators, n(%)	11 (26.2)	5 (23.8)	6 (28.6)

ACEI- angiotensin converting enzyme inhibitor; ARB- angiotensin receptor blocker; BMI- body mass index; DBP- diastolic blood pressure; SBP- systolic blood pressure; *statistically significant difference compared to non-responder (p < 0.01); ^#^statistically significant difference compared to non-responder (p < 0.001); ^§^statistically significant difference compared to the number of antihypertensives at baseline (p < 0.01).

During RDN 9.9 ± 3.6 ablations were performed. As shown in Table 2 there was no statistically significant difference in procedure-related parameters such as the diameter of renal arteries, the distance between the origin of the renal artery and the last ablation point, the number of ablation points, or the anatomy of renal arteries.

**Table 2:** Renal denervation procedure characteristics.

	Non-responder n = 21	Responder n = 21	p-value
Standard anatomy of renal arteries, n(%)*	11(52.4)	15(71.4)	ns
Sum of the ablation points	10.4 ± 4.2	9.3 ± 2.7	ns
Diameter of the right renal artery, mm	7.0 ± 1.5	7.1 ± 1.1	ns
Diameter of the left renal artery, mm	7.1 ± 1.4	7.3 ± 1.1	ns
Distance ostium RRA-last ablation point, mm	39.5 ± 14.5	39.7 ± 13.0	ns
Distance ostium LRA-last ablation point, mm	37.6 ± 9.1	40.3 ± 12.4	ns

*Standard anatomy, considered when single renal artery without any anatomical abnormalities on each site is present; LRA- left renal artery; RRA- right renal artery.

**Figure 1:**
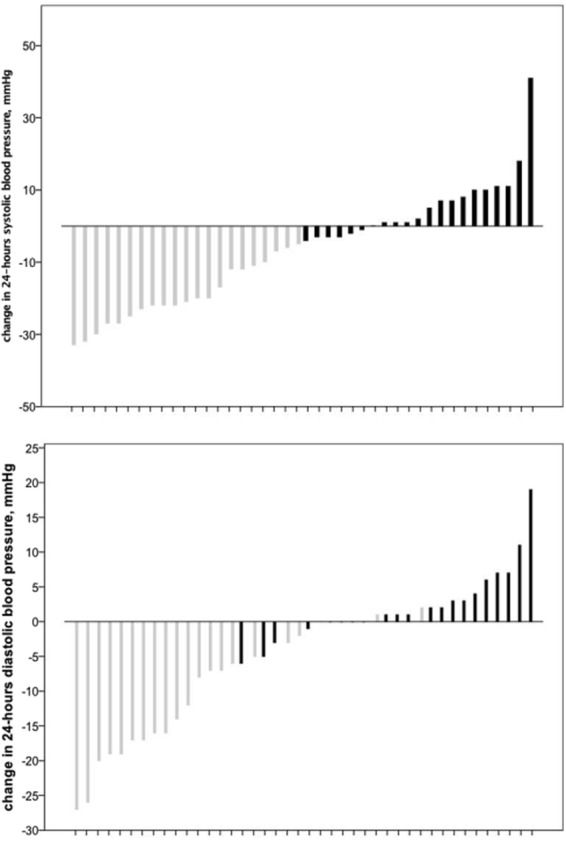
Individual changes in 24 h systolic and diastolic blood pressure from baseline to 12 months after renal denervation; grey bars represent responder and black bars represent non-responder.

**Figure 2:**
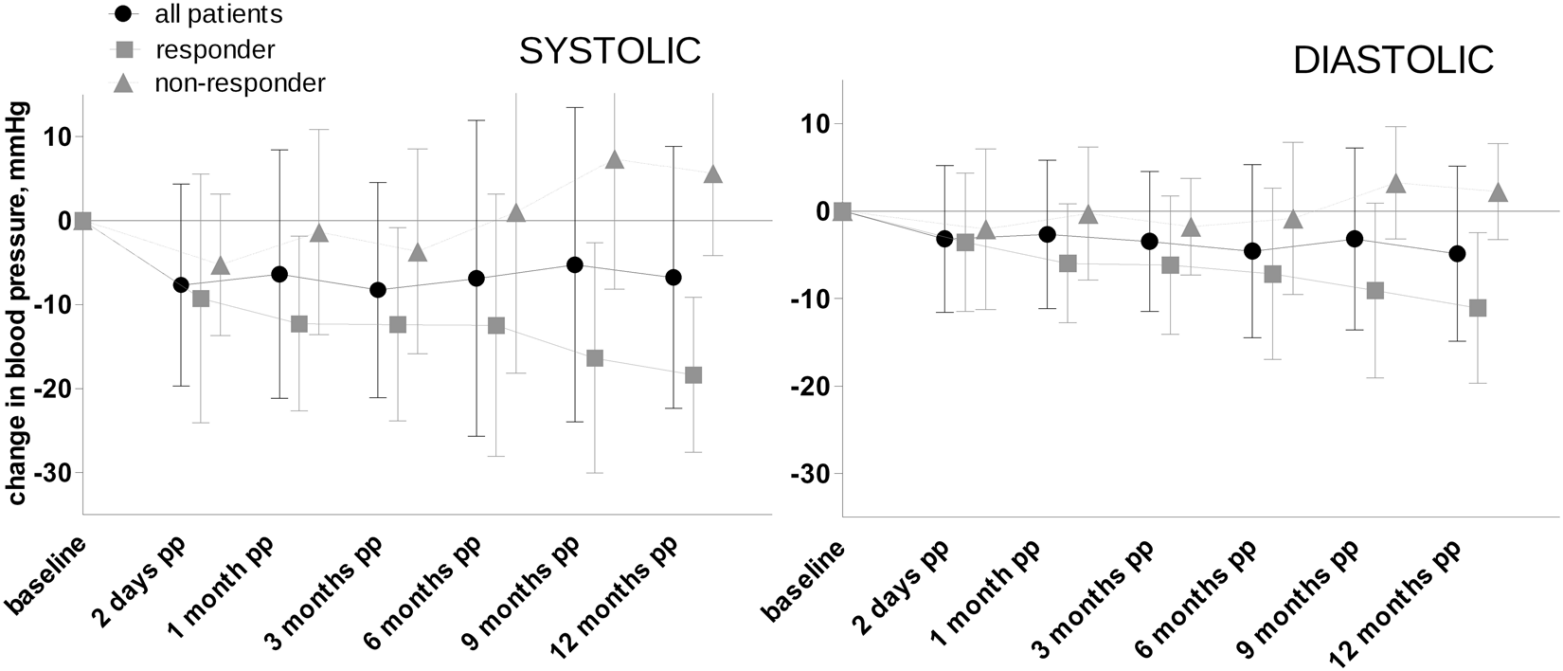
Mean changes in 24 h ambulatory systolic and diastolic blood pressure at 2 days, 1, 3, 6, 9 and 12 months after RDN (pp- post procedure); whiskers represent standard deviation.

We observed a significant decline in mean 24 h-SBP and 24 h-DBP in the whole group and the responder group twelve months after RDN. Non-responder did not show any significant change in 24 h-SBP and 24 h-DBP. Details for whole group, responder and non-responder are shown in Table 3. Individual changes in 24-h mean SBP and DBP from baseline to twelve months are presented in Fig. 1. 24 h-SBP and -DBP mean changes at two days, one month, three months, six months, nine months and twelve months after RDN are shown in Fig. 2.

**Table 3:** 24-hours systolic (SBP) and diastolic (SBP) blood pressure at baseline and twelve months after renal denervation.

	Baseline	Twelve months after RDN	p-value
24-hours SBP (mm Hg)			
Whole collective	149.8 ± 13.3	141.2 ± 14.6	0.001
Responder	155.1 ± 11.4	135.0 ± 14.6	<0.001
Non-responder	144.5 ± 13.1	147.4 ± 11.8	0.26
24-hours DBP (mm Hg)			
Whole collective	83.3 ± 11.7	78.8 ± 11.2	0.007
Responder	89.5 ± 9.1	78.1 ± 9.1	<0.001
Non-responder	77.0 ± 10.8	79.5 ± 13.1	0.052

**Figure 3:**
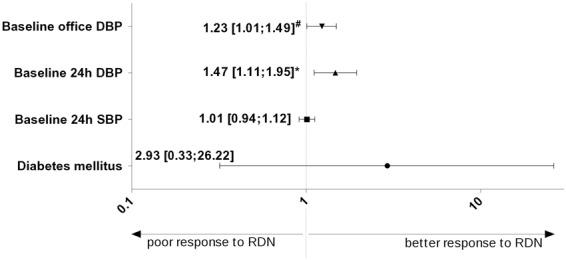
Impact of diabetes mellitus, baseline 24 h mean systolic blood pressure (SBP) and baseline 24 h mean diastolic blood pressure (DBP) on the response to renal denervation assessed with binary logistic regression analysis. X-axis represents odds ratio with logarithmic (log10) scale; *statistically significant with p = 0.007; ^#^statistically significant with p = 0.045. Variables entered in multivariate analysis: baseline office SBP and DBP, baseline mean 24 h-SBP and mean 24 h-DBP, diabetes, isolated systolic hypertension, baseline heart rate, age, sex, body-mass-index, estimated glomerular filtration rate.

In the univariate analysis significantly different parameters between responder and non-responder group (see Table 1) and age, sex, BMI, baseline office SBP and DBP, eGFR, heart rate, and ISH were inputted in a binary logistic regression. For prediction of response to RDN the odds ratio of baseline office DBP and 24 h-ABPM DBP was 1.2 [95% CI 1.0 to 1.5; p = 0.045] and 1.5 [95% CI 1.1 to 1.9; p = 0.007] respectively; the odds ratio of baseline 24 h-ABPM SBP and diabetes mellitus were 1.01 [95% CI 0.9 to 1.1, non-significant] and 2.9 [95% CI 0.3 to 26.2, non-significant] (Fig. 3). We also performed multiple linear regression analysis for change in mean 24 h-SBP and 24 h-DBP including the same independent variables as in the analysis of predictive effect based on defined response cut-off (>10 mm Hg change in office SBP). In this analysis, baseline mean 24 h-DBP predicted significantly decrease in mean 24 h-SBP (p = 0.003) and mean 24 h-DBP (p = 0.001) after RDN.

**Figure 4:**
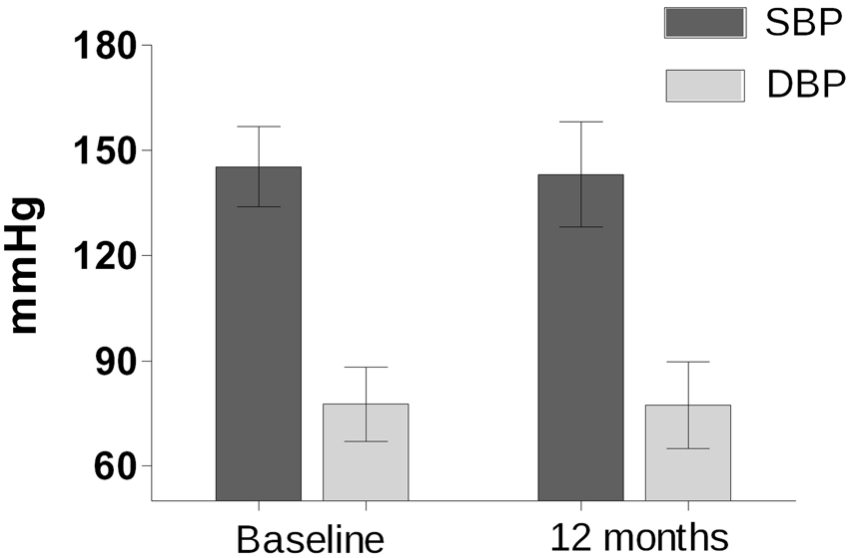
24-hours systolic (SBP) and diastolic (DBP) blood pressure in diabetic patients at baseline and 12 months after renal denervation; bars show means and whiskers show standard deviation.

The particular analysis of diabetic patients showed significantly higher baseline mean 24 h-SBP (154.3 ± 13.7 vs. 145 ± 11.5 mm Hg, p = 0.001) and mean 24 h-DBP (88.9 ± 10.0 vs. 77.6 ± 10.6 mm Hg, p = 00.1) compared to non-diabetics. Both mean 24 h-SBP and 24 h-DBP did not change significantly twelve months after RDN in diabetic patients (Fig. 4). Prevalence of isolated systolic hypertension was 33% in non-diabetics and 67% in diabetic patients (p = 0.06).

## Discussion

In this study, we performed a retrospective analysis regarding the BP-lowering effect of RDN-procedure in our center. We saw a clinically relevant reduction in 24 h mean SBP (8.6 mm Hg) and mean DBP (4.5 mm Hg). Observed amount of BP-reduction is comparable with the findings of recent randomized sham-controlled trials, which were explicitly designed to eradicate known limitations of RDN trials^8–10^. In addition to the inclusion of the sham procedure and assessment of BP-lowering effect based on results of ABPM, investigators also performed RDN in subjects without any antihypertensive medication (OFF MED approach) to assess the real effect of RDN on BP. Furthermore, in the case of electrical RDN, the ablation procedure was performed with the latest generation multielectrode catheter and generator^8,9^. Acknowledging the sophisticated trial design and improvements in ablation techniques, our patients had similar BP response after RDN compared to patients from above mentioned modern RDN-trials. This circumstance underlines the reliability of our data. Worth mentioning, baseline 24 h-DBP in SPYRAL HTN-ON and -OFF MED trials was substantially higher than in our analysis.

We choose to dichotomize the BP-response after RDN and set the cut-off by ≥10 mm Hg fall in office SBP as already done in previous studies^12,13^. We observed a statistically significant independent impact of baseline office DBP and mean 24 h-DBP on response to RDN using multiple binary regression analysis. Significant predictive effect of mean baseline 24-h DBP also remained, when the BP-lowering effect was assessed as a continuous variable using multiple linear regression analysis.

Virtually all previously published RDN studies identified baseline BP as the major primary determinant of BP response after RDN: Symplicity HTN-1 Investigators reported the issue for office BP response already in the initially published results^14^. Randomized sham-controlled SIMPLICITY HTN 3 trial confirmed these findings. Various registry analyses including experiences from different countries also found baseline BP as a major predictor of BP-lowering effect after RDN and confirmed data coming from proof-of-concept studies and randomized trials by the evidence from real-life setting^15–21^. Noteworthy, baseline SBP predicted lowering effect in SBP and baseline DBP predicted lowering effect in DBP in all previously mentioned studies. In contrast, we observed baseline office DBP and 24 h-DBP being predictors for the fall in SBP (office and 24 h-ABPM). To our knowledge, this finding has not been reported yet and thus extends available evidence. A possible biological explanation could be that higher DBP might reflect arterial vessels without severe and irreversible wall damage. Profound arteriosclerosis leads to increased arterial stiffness, higher pulse wave velocity (PWV), lower DBP and increased prevalence of isolated systolic hypertension (ISH)^22^. In this case, sympathetic overdrive is probably not a dominant driving mechanism of refractory arterial hypertension and thus associated with impaired response to RDN. Growing evidence supports this hypothesis that patients with advanced arterial stiffness and thus severe arterial wall damage as indicated by increased PWV^23^, aortic calcifications^24^ or ISH^16,25^ showed attenuated BP response after RDN.

One of the major causes of arteriosclerosis is diabetes mellitus. However, the impact of diabetes mellitus on BP response failed the statistical significance in multiple regression analysis, though it has shown a statistically significant negative impact on the BP response after RDN in univariate analysis among our patients. Further analysis of the subgroups with and without diabetes mellitus revealed that the former had significantly higher mean baseline SBP and DBP in ABPM, which could attenuate the effect of diabetes mellitus in the statistical analysis. Furthermore, patients with diabetes had also numerically more ISH compared to the group without diabetes. Lower baseline DBP and higher prevalence of ISH in non-responders and diabetic patients could be an expression of increased arterial stiffness and thus poorer response to RDN in our collective.

One of the strengths of our analysis is that the response to RDN was assessed with office BP measurements and ABPM, though patients were classified as responder or non-responder according to the amount of office SBP change. However, grouping in responder and non-responder according to office BP response reflected the response of 24-h ambulatory SBP and DBP in our analysis very well. As already shown, office BP might have some potential confounding characteristics such as “regression to the mean” phenomenon and white coat hypertension, which significantly influence the amount of BP change over time^8^. These phenomena could be in part eliminated by ABPM^11^.

We acknowledge several limitations of our analysis. Our findings were obtained from a single centre and analysed in a retrospective fashion. Thus, looking for potential predictors of response to RDN our findings reflect associations and can only generate a possible hypothesis, which should then be tested in appropriately designed prospective studies. Relatively small number of the included patients and open non-blinded design of the analysis might not be able to cover the abundance of confounding factors in RDN as we have learned from previous studies. Keeping the potentially higher risk of confounding in mind, our results show a similar response to RDN compared to recent second-generation sham-controlled randomized studies^8–10^.

Testing patient compliance was not a part of clinical routine during the follow up after the RDN in our collective. Thus, confounding of the BP-lowering effect of RDN by varying medication intake cannot be ruled out. It is a common problem of all RDN studies or analyses, which included patients on antihypertensive medications without compliance testing as an inclusion criterion or as a part of follow up. We cannot distinguish the true resistant hypertensive patients from the patients who are incompliant to their antihypertensive drugs. Among the non-responders in our study collective, the BP increased over time. A possible explanation of this finding could be the higher amount of incompliant patients, who took their medication in preparation to RDN and shortly after and then reduced or stopped it.

The cut-off margin for responder was defined for office BP based on previous studies^18^. Study findings could thus not be generally translated to response in ABPM and other cut-offs for a definition of responder vs. non-responder to RDN. Hence, we additionally looked for response predictors for change in mean 24 h-SBP and mean 24 h-DBP in ABPM and could demonstrate that higher baseline 24-DBP, but not baseline office DBP, significantly predicted a higher decrease of mean 24 h-SBP and 24 h-DBP 12 months after RDN. In our analysis, we showed the trend for diabetes mellitus to be a predictor for negative RDN response, which failed to remain statistically significant in the multiple logistic regression. This could be due to a relatively small study collective and further prospective studies directly approaching this issue with more patients are needed.

Recently released new guidelines on the management of hypertension by European Society of Hypertension and European Society of Cardiology do not recommend to use RDN in the clinical routine as hypertension treatment method anymore. One of the concerns leading to this recommendation was insufficient data about the suitable subpopulation of hypertensive patients and lack of predictors for RDN response^26^.

The primary conclusion and clinical implication from our study are that DBP, measured in office and with ABPM, seems to be a predictor of response to RDN. Given that lower DBP could be a reflection of increased arterial stiffness and more advanced arterial wall damage, our data add to the available evidence pointing to impaired response to RDN in patients with increased arterial stiffness. Further properly designed randomized trials are warranted to confirm this finding as well as further investigate the role of diabetes mellitus and arterial stiffness in RDN.

## Methods

This retrospective analysis was performed according to the Declaration of Helsinki and written informed consent was obtained from all patients included. The local ethics committee (ethics committee of Charité Medical University Berlin, Germany) approved the study. There is no restriction on the availability of additional materials or information, which can be any time obtained by sending a query to the corresponding author via email.

Patients were considered to have resistant hypertension, when the mean of three measurements of office systolic blood pressure was >140 mm Hg and/or of office DBP >90 mm Hg respectively and the mean SBP >130 mm Hg and mean DBP >80 mm Hg with 24 hours ambulatory blood pressure monitoring. 81 patients with resistant hypertension underwent RDN between November 2010 and April 2014. All of them were treated with at least three antihypertensive drugs including a diuretic at maximally tolerated dosages before performing the RDN. Common secondary causes of hypertension such as sleep apnea syndrome, hemodynamic significant renal artery stenosis, hyperaldosteronism, hypercortisolism, abnormality of thyroid function and pheochromocytoma were excluded prior to RDN.

Further exclusion criteria were pregnancy, age below 18 years, implanted peacemaker or intracardiac defibrillators, severe cardiac valve abnormalities and renal artery diameter <4 mm or length of renal artery <20 mm. Renal artery anatomy was checked before intervention by either CT-angiography or MR-angiography.

For RDN, the Symplicity RDN Catheter System (Medtronic Inc., Minneapolis, Minnesota, USA) was used via the right femoral approach. RDN procedure was performed in both renal arteries during the same session with a minimum of five ablation points in each renal artery. The RDN procedure was performed under general anesthesia. We defined the bilateral presence of single renal artery without any anatomical abnormalities as “standard anatomy”; any other case was defined as “non-standard” anatomy.

Routine clinical follow-up visits were performed after two days, one month, three months, six months, nine months, and twelve months. During each visit routine blood tests including assessment of kidney function and albuminuria were taken, information about current medication was collected and office blood pressure measurement, as well as ABPM were performed. ABPM was performed using Spacelabs 90207-2 device (Spacelab Healthcare, Snoqualmie, WA 98065 US). The interval between 0600 to 2200 was defined as daytime (readings every 20 minutes) and the interval between 2200 and 0600 was defined as night-time (readings every 30 minutes). The cuff was placed on the non-dominant upper arm and patients were instructed to keep their arm calm during the measurement. 42 patients with complete data for ABPM readings at baseline and after 12 months were included in this retrospective analysis.

Whenever a reduction in office SBP achieved ≥10 mm Hg after twelve months compared to baseline, the patient was considered to be a responder to RDN.

Isolated systolic hypertension was defined as the presence of SBP >140 mm Hg with simultaneously DBP ≤90 mm Hg detected with office measurements or with ABPM.

### Statistical Analysis

Continuous variables are expressed as mean with standard deviation. Presence of normal distribution was assessed with the Kolmogorov Smirnov test. In case of normal distribution, we used the t-test for comparison. In case of non-normal distribution, we used the Mann-Whitney-U test for comparison.

Discrete parameters are expressed as the number of patients and percentages. The differences were compared using **χ**^**2**^-test.

A multiple binary logistic regression analysis with stepwise forward inclusion model was applied to determine the predictors of RDN response after twelve months.

The level of significance used for all tests was a two-sided p-value of 0.05.
