# Author Correction: Predictors for success in renal denervation–a single centre retrospective analysis

**DOI:** 10.1038/s41598-019-48857-z

**Published:** 2019-08-22

**Authors:** Alexander Reshetnik, Christopher Gohlisch, Christian Scheurig-Münkler, Maximilian De Bucourt, Walter Zidek, Markus Tölle, Markus van der Giet

**Affiliations:** 1 Charité – Universitätsmedizin Berlin, a corporate member of Freie Universität Berlin, Humboldt-Universität zu Berlin, and Berlin Institute of Health, Campus Benjamin Franklin, Department of Nephrology, Hindenburgdamm 30, 12203 Berlin, Germany; 2 Charité – Universitätsmedizin Berlin, a corporate member of Freie Universität Berlin, Humboldt-Universität zu Berlin, and Berlin Institute of Health, Campus Benjamin Franklin, Department of Radiology, Hindenburgdamm 30, 12203 Berlin, Germany; 3 Department for Diagnostic and Interventional Radiology and Neuroradiology, Universitaetsklinikum Augsburg, Stenglinstr. 2, 86156 Augsburg, Germany

Correction to: *Scientific Reports* 10.1038/s41598-018-33783-3, published online 19 October 2018

Christian Scheurig-Münkler was omitted from the author list in the original version of this Article. This has been corrected in the PDF and HTML versions of the Article.

The Author Contributions section now reads:

Dr. Alexander Reshetnik: conception of study design, analysis and interpretation of data, drafting the article.

Dr. Christopher Gohlisch: writing the article; acquisition of data.

Dr. Christian Scheurig-Münkler: acquisition of data; interpretation of data, providing intellectual content of critical importance to the work described; final approval of the version to be published.

Dr. Maximilian De Bucourt: interpretation of data; providing intellectual content of critical importance to the work described; final approval of the version to be published.

Prof. Dr. Walter Zidek: providing intellectual content of critical importance to the work described; final approval of the version to be published.

Dr. Markus Tölle: providing intellectual content of critical importance to the work described; final approval of the version to be published.

Prof. Dr. Markus van der Giet: conception of study design, interpretation of data; revising the article.

